# Supplementary material for: Emissions Reduction Strategies for the Orange and Cherry Industries in New South Wales
Source: Foods. 2023 Sep 5;12(18):3328. doi: 10.3390/foods12183328 (PMC10527705; doi:10.3390/foods12183328)
Supplement: Supplementary file 1 [file foods-12-03328-s001.zip › foods-2539606-supplementary.pdf]

Supplementary materials

Emissions Reduction Strategies for the Orange and Cherry Industries in New South Wales

Aaron T. Simmons [1,2,*], Marja Simpson [3], Paul-Antoine Bontinck [4], John Golding [5], Tim Grant [4], Jess Fearnley [3] and Steven Falivene [6]

1 NSW Department of Primary Industries, Muldoon St., Taree, NSW 2430, Australia
2 School of Business, University of New England, Elm Ave, Armidale, NSW 2350, Australia
3 NSW Department of Primary Industries, Orange Agricultural Institute, Orange, NSW 2800, Australia; marja.simpson@dpi.nsw.gov.au (M.S.); jessica.fearnley@dpi.nsw.gov.au (J.F.)
4 Lifecycles, 2/398 Smith Street, Collingwood, VIC 3066, Australia; paul-antoine@lifecycles.com.au (P.-A.B.); tim@lifecycles.com.au (T.G.)
5 NSW Department of Primary Industries, Locked Bag 26, Gosford, NSW 2250, Australia; john.golding@dpi.nsw.gov.au
6 NSW Department of Primary Industries, P.O. Box 62, Dareton, NSW 2717, Australia; steven.falivene@dpi.nsw.gov.au
* Correspondence: aaron.simmons@dpi.nsw.gov.au

Table S1: Average yield, orchard activities and inputs for a cherry orchard in Orange NSW

**Cherry orchard activities**	**unit**	**Year 1**	**Years 2 and 3**	**Years 4 and 5**	**Years 6+**
average yield	t/ha	0	1.5	8.5	15
electricity for pumping for irrigation	ML/ha	0	2.9	2.9	2.9
inter-row cultivation	times/ha	0	2	2	2
inter-row tractor operation	times/ha	0	4	4	4
air blast spraying	times/ha	0	11	11	11
boom spraying	times/ha	0	4	4	4
ammonium nitrate	kg/ha	0	8	8	8
calcium ammonium nitrate	kg/ha	0	266	280	525
diammonium phosphate	kg/ha	0	2.7	2.7	2.7
potassium nitrate	kg/ha	0	177	187	350
monoammonium phosphate	kg/ha	0	13.5	13.5	13.5
benomy	**kg/ha**	**0**	**0.5**	**0.5**	**0.5**
copper oxychloride	kg/ha	0	1.925	1.925	1.925
gibberelic acid	kg/ha	0	0.28737	0.28737	0.28737
tau-fluvalinate	kg/ha	0	0.082	0.082	0.082
mancozeb	kg/ha	0	2.25	2.25	2.25
ziram	kg/ha	0	2.28	2.28	2.28
horticultural oil	kg/ha	0	25.8	25.8	25.8
undertree irrigation system	times/ha	1	0	0	0
levelling	times/ha	1	0	0	0
offset disc harrowing	times/ha	1	0	0	0
tree removal	times/ha	1	0	0	0
raking tree residues	times/ha	2	0	0	0
row mounding	times/ha	1	0	0	0
seedling transplanting	times/ha	1	0	0	0
soil cultivation	times/ha	1	0	0	0
soil ripping	times/ha	1	0	0	0
stump pulling	times/ha	1	0	0	0
transport of inputs	kg/km/ha	0	100 000	105 000	187 000

Table S2: Average yield, orchard activities and inputs for an orange orchard in the Sunraysia region of NSW

**orange orchard activities**	**unit**	**Year 1**	**Year 2 to 4**	**Year 5 to 7**	**Year 8 to 10**	**Years 10+**
average yield	t/ha	0	1	8	25	40
electricity for pumping for irrigation	ML/ha	0	4.67	6.67	9	10
air blast spraying	times/ha	0	10	6	2	5
fertiliser side dressing	times/ha	0	0	0	10	2
fertiliser spreading	times/ha	0	3	10	2	10
boom spraying	times/ha	0	3.3	4.3	4	5.3
ammonium nitrate	kg/ha	0	24.2	24.2	43.5	38.5
ammonium sulphate	kg/ha	0	0.606	0.757	0.881	0.549
calcium ammonium nitrate	kg/ha	0	14	18.7	18.8	9.5
diammonium phosphate	kg/ha	0	0.156	0.156	0.28	0.248
double superphosphate	kg/ha	0	0.884	1.13	1.25	0.734
monoammonium phosphate	kg/ha	0	25.2	25.2	75.3	30.2
potassium chloride	kg/ha	0	0.155	0.155	0.279	0.248
potassium nitrate	kg/ha	0	28.2	30.2	150	85.3
carfentrazone-ethyl	kg/ha	0	0.0012	0.0012	0.0012	0.0012
chlorpyrifos	kg/ha	0	0.405	0.405	0.96	1.92
diquat	kg/ha	0	0.0012	0.0012	0.345	0.345
gluufosinate ammonium	kg/ha	0	0	0	0.03	0.03
glyphosate	kg/ha	0	1.4	1.4	1.4	1.4
haloxyfop	kg/ha	0	0.416	0.416	0.416	0.416
imidacloprid	kg/ha	0	0.416	0.416	1.02	1.02
methiocarb	kg/ha	0	0.405	0.405	7.5	7.5
paraquat	kg/ha	0	0.405	0.405	0.405	0.405
potassium sulphate	kg/ha	0	0.155	0.155	0.279	0.248
single superphosphate	kg/ha	0	0.302	0.351	0.475	0.346
spinetoram	kg/ha	0	0.416	0.416	3.85	5.8
triple superphosphate	kg/ha	0	0.199	0.214	0.338	0.277
urea	kg/ha	0	8.96	8.96	9.08	9.05
cultivating	times/ha	1	0	0	0	0
levelling - soil cultivation	times/ha	1	0	0	0	0
offset disc harrowing	times/ha	1	0	0	0	0
orchard raking	times/ha	2	0	0	0	0
ripping - soil cultivation	times/ha	1	0	0	0	0
seedling transplanting	times/ha	1	0	0	0	0
stump pulling	times/ha	1	0	0	0	0
tree removal	times/ha	1	0	0	0	0
undertree irrigation system	times/ha	1	0	0	0	0
transport, truck, 28t	kg/km/ha	0	52300	58600	155000	93700
